# SIRT1 facilitates hepatocellular carcinoma metastasis by promoting PGC-1α-mediated mitochondrial biogenesis

**DOI:** 10.18632/oncotarget.8711

**Published:** 2016-04-12

**Authors:** Yuming Li, Shangcheng Xu, Jing Li, Lu Zheng, Min Feng, Xiaoya Wang, Keqiang Han, Huifeng Pi, Min Li, Xiaobing Huang, Nan You, Yewang Tian, Guohua Zuo, Hongyan Li, Hongzhi Zhao, Ping Deng, Zhengping Yu, Zhou Zhou, Ping Liang

**Affiliations:** ^1^ Department of Hepatobiliary Surgery, Second Affiliated Hospital of Third Military Medical University, Chongqing 400037, China; ^2^ Department of Occupational Health, Third Military Medical University, Chongqing 400038, China; ^3^ Department of Military Nursing, School of Nursing, Third Military Medical University, Chongqing 400038, China

**Keywords:** SIRT1, PGC-1α, mitochondrial biogenesis, metastasis, hepatocellular carcinoma

## Abstract

SIRT1 is a multifaceted NAD^+^-dependent protein deacetylase known to act as a tumor promoter or suppressor in different cancers. Here, we describe a novel mechanism of SIRT1-induced hepatocellular carcinoma (HCC) metastasis. SIRT1 overexpression was frequently detected in human HCC specimens and was associated with microvascular invasion (*P* = 0.0039), advanced tumor node metastasis (TNM) stages (*P* = 0.0016), HCC recurrence (*P* = 0.021) and poor outcomes (*P* = 0.039). Lentivirus-mediated knockdown of SIRT1 in MHCC97H cells reduced invasion and metastasis *in vitro* and *in vivo*. SIRT1 depletion attenuated mitochondrial biogenesis and adenosine triphosphate (ATP) production but did not affect epithelial-mesenchymal transition. Elevated SIRT1 expression strongly correlated with the upregulation of PGC-1α in HCC specimens, and ectopic expression of SIRT1 increased PGC-1α levels. In cell assays and an orthotopic transplantation model, PGC-1α overexpression reversed the inhibitory effects of SIRT1 depletion on invasion and metastasis by enhancing mitochondrial biogenesis. These findings reveal the involvement of SIRT1 in HCC metastasis and provide a rationale for exploring therapeutic targets against the SIRT1/PGC-1α axis.

## INTRODUCTION

Hepatocellular carcinoma (HCC) is the fifth most common cancer malignancy and the third leading cause of cancer-related mortality [1]. As most HCC patients are diagnosed in advanced stages and have intrahepatic and extrahepatic metastases, the prognosis is extremely poor for HCC patients: the five-year postoperative survival rate is only 30%–40%, due to the high frequency of tumor recurrence and distant metastasis [2]. Therefore, a thorough understanding of the metastatic mechanism is vital for the development of efficacious therapeutics.

Sirtuins, a highly conserved protein family that are homologous to silent information regulator 2 (SIR2) [3], are nicotinamide adenine dinucleotide (NAD^+^)-dependent histone deacetylases that regulate lifespan and aging in mammals [4, 5]. Seven homologs of SIR2 (SIRT1-7) have been identified. Of these, SIRT1 is predominantly localized to the nucleus and functions in energy metabolism, stress responses, cell survival, telomere maintenance, and genomic stability [6–8]. Currently, the role of SIRT1 in cancer is controversial, due to the Janus-faced activity of SIRT1 in tumorigenesis [9]. Several reports have shown that SIRT1 acts as a tumor promoter: SIRT1 enhanced the migration of melanoma cells by regulating lamellipodial extension [10], and cooperated with ZEB1 to induce the epithelial-mesenchymal transition (EMT) and increase prostate cancer cell migration and invasion [11]. However, a few studies have classified SIRT1 as a negative regulator of tumor progression, as SIRT1 upregulation inhibited the migration and metastasis of oral squamous cell carcinoma cells through the deacetylation of SMAD4, which suppressed the effects of Transforming Growth Factor beta (TGF-β) signaling [12]. Although recent studies have demonstrated that SIRT1 overexpression promotes HCC tumorigenesis and is essential for telomere maintenance [8, 13], the precise mechanism whereby SIRT1 impacts HCC metastasis is still ambiguous.

Metabolic reprogramming is a vital feature of invading tumor cells and has emerged as an attractive target for novel therapeutic strategies [14]. The mitochondria provide the majority of the energy for most cells, as they synthesize ATP via oxidative phosphorylation [15]. Recently, enhanced mitochondrial biogenesis and oxidative phosphorylation were shown to be critical in promoting cancer metastasis [16]. The peroxisome proliferator–activated receptor γ coactivator 1α (PPARGC1A, hereafter abbreviated as PGC-1α) is a member of a small family of transcriptional coactivators that promote mitochondrial biogenesis and respiration [17, 18]. PGC-1α is implicated in mitochondrial biogenesis, thermogenesis, adipocyte differentiation and gluconeogenesis by interacting with a number of nuclear receptors, such as nuclear respiratory factor-1 (NRF-1), glucocorticoid receptor (GR), and hepatocyte nuclear factor 4α (HNF4α) [19–21]. PGC-1α expression also enhanced the invasion and migration of breast cancer and melanoma cells [16]. However, the effects of PGC-1α on migration and metastasis in HCC have not yet been described.

PGC-1α is a key downstream target of SIRT1. SIRT1 physically and directly interacts with PGC-1α and the two proteins co-immunoprecipitate as a molecular complex. [22]. AdipoR1 was shown to activate SIRT1, which increased the mitochondrial content and enzymes by upregulating PGC-1α expression in skeletal muscle [23]. Recently, the interaction between SIRT1 and PGC-1α has attracted much attention in the study of tumorigenesis, as a SIRT1/PGC-1α-dependent increase in oxidative phosphorylation enhanced the resistance of colon cancer to chemotherapy [24]. Adiponectin inhibited pancreatic cancer cell apoptosis by activating AMPK/SIRT1/PGC-1α signaling [25].

However, it remains unknown whether SIRT1/PGC-1α signaling is involved in HCC metastasis. In this study, we demonstrated that SIRT1 overexpression was frequently detected in HCC specimens and significantly enhanced migration and metastasis in HCC *in vitro* and *in vivo*. Therefore, we hypothesized that SIRT1 facilitates HCC metastasis by activating PGC-1α, thus enhancing mitochondrial biogenesis and altering energy metabolism.

## RESULTS

### SIRT1 expression was elevated in HCC cell lines and tissues and predicted poor prognosis in HCC patients

**Figure 1:**
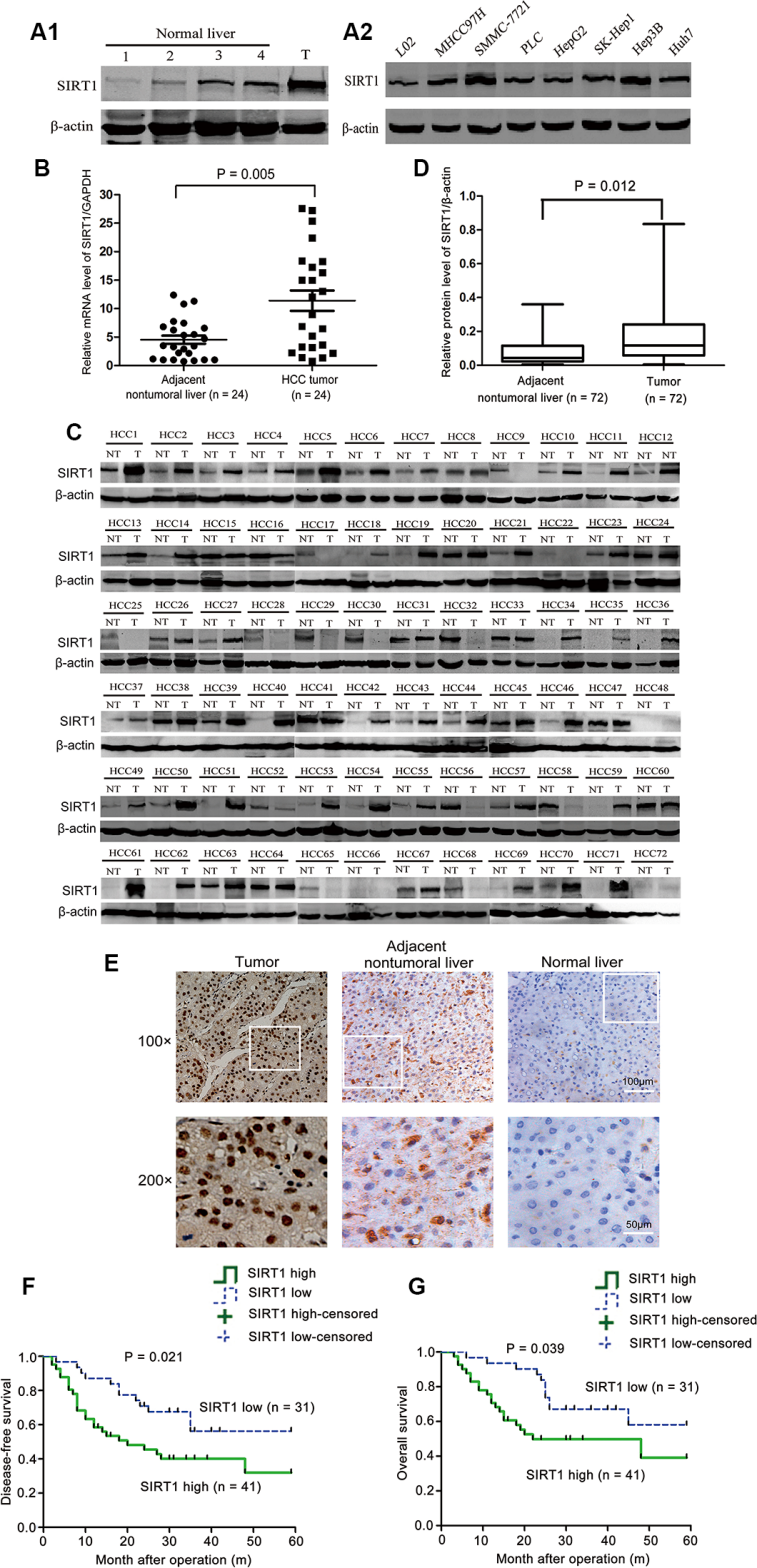
SIRT1 expression was elevated in HCC cell lines and tissues and predicted poor prognosis in HCC patients (**A1**, **A2**) Western blot analysis of SIRT1 expression in normal livers, HCC tumor specimens, the immortalized human liver cell line LO2 and seven different hepatoma cell lines. The normal livers samples were obtained from patients who did not have HCC or hepatitis. T, tumor tissues from HCC patients. (**B**) The relative levels of *SIRT1* mRNA were significantly greater in HCC tumors than in adjacent nontumoral liver tissues (*n* = 24) (**P = 0.005). (**C**) Western blot analysis of SIRT1 expression in HCC tumors compared to the paired adjacent nontumoral liver tissue (NT, adjacent nontumoral liver; T, tumor). β-actin was used as the internal standard for equal protein loading. (*n* = 72 patients). (**D**) The relative levels of SIRT1 protein were determined by normalization of the SIRT1 density to the β-actin density. SIRT1 protein levels were significantly greater in HCC tumors than in adjacent nontumoral liver tissues (*n* = 72) (*P = 0.012). (**E**) Representative immunohistochemical stainings of SIRT1 expression in paired primary HCC tumor, adjacent nontumoral liver and normal liver samples. (Original magnification ×100, bar = 100 μm. Designated areas with higher magnification ×200, bar = 50 μm). (**F**, **G**) Kaplan-Meier analysis indicating the correlation of SIRT1 overexpression with the shorter disease-free survival time (*P* = 0.021) and worse overall survival rate (*P* = 0.039) of HCC patients.

To explore the oncogenic role of SIRT1 in HCC, we first determined the expression of SIRT1 using several human liver tissues and a panel of HCC cell lines. As shown in Figure 1A1, SIRT1 expression was low in normal liver tissues but high in HCC specimens. SIRT1 expression was also elevated in HCC cell lines compared to L02 cells (an immortalized human liver cell line) (Figure 1A2). To better determine the expression pattern of SIRT1 in HCC, we also evaluated SIRT1 levels in additional clinical specimens from HCC patients. The levels of the *SIRT1* transcripts were significantly greater in HCC tissues than in adjacent nontumoral liver tissues (Figure 1B). In addition, among 72 HCC specimens, SIRT1 protein was frequently upregulated in HCC tissues compared to paired adjacent nontumoral liver tissues (Figure 1C, 1D). Overexpression of SIRT1 (defined as a > 2-fold increase compared to the corresponding nontumoral tissues) was detected in 56.9% (41/72) of HCC tumors (Figure 1C). Immunohistochemical (IHC) analyses revealed that SIRT1 was primarily localized to the nucleus and was highly expressed in HCC tumors compared to adjacent nontumoral tissues and normal liver tissues (Figure 1E).

We next determined the correlations between SIRT1 expression and various clinical parameters to investigate the clinical significance of SIRT1 expression in HCC. The clinicopathological parameters of HCC patients are summarized in Table 1. Increased SIRT1 expression in HCC patients correlated with the incidence of portal vein tumor thrombus (*P* = 0.0039) and advanced tumor stages (*P* = 0.0016), but not with the other clinicopathological features listed in Table 1. HCC patients with overexpression of SIRT1 had shorter disease-free survival (*P* = 0.021) and worse overall survival (*P* = 0.039) than patients without SIRT1 overexpression (Figure 1F, 1G). Thus, SIRT1 overexpression could serve as a valuable index for predicting disease recurrence and poor survival in HCC patients.

**Table 1:** Correlative analysis of SIRT1 protein levels with clinicopathological features

clinical variables	No.of specimens	^a^Relative level of STRT1 expression		*P* Value
		low	high	
Age, mean ± SD, y	72	51.1 ± 10.3	49.4 ± 11.6	NS^c^
Sex				
Male	65	28	37	NS
Female	7	3	4	
Etiology				
HBV	61	25	36	NS
Non-HBV	11	6	5	
Child–Pugh class				
A	70	30	40	NS
B	2	1	1	
Cirrhosis				
with	59	23	36	NS
without	13	8	5	
AFP, ng/mL				NS
< 20	26	12	14	
20–100	10	4	6	
100–400	9	5	4	
> 400	27	10	17	
Portal vein tumor thrombus				0.0039
without	46	26	20	
with	26	5	21	
Tumor size, cm				NS
< 3	10	5	5	
3–5	18	11	7	
> 5	44	15	29	
TNM^b^				
Stage I	44	26	18	0.0016
Stage II or III	28	5	23	
Edmondson–Steiner grade				NS
I or II	29	14	15	
III or IV	43	17	26	
Milan criteria				NS
Within	18	12	6	
Beyond	54	19	35	

a High tumoral SIRT1 expression was regarded at > 2-fold up-regulation, relative to the adjacent nontumoral liver;

b Sixth edition of UICC TNM staging system of HCC (2002).

c NS: no significant difference.

### Effect of SIRT1 knockdown on HCC cell proliferation and tumorigenicity

**Figure 2:**
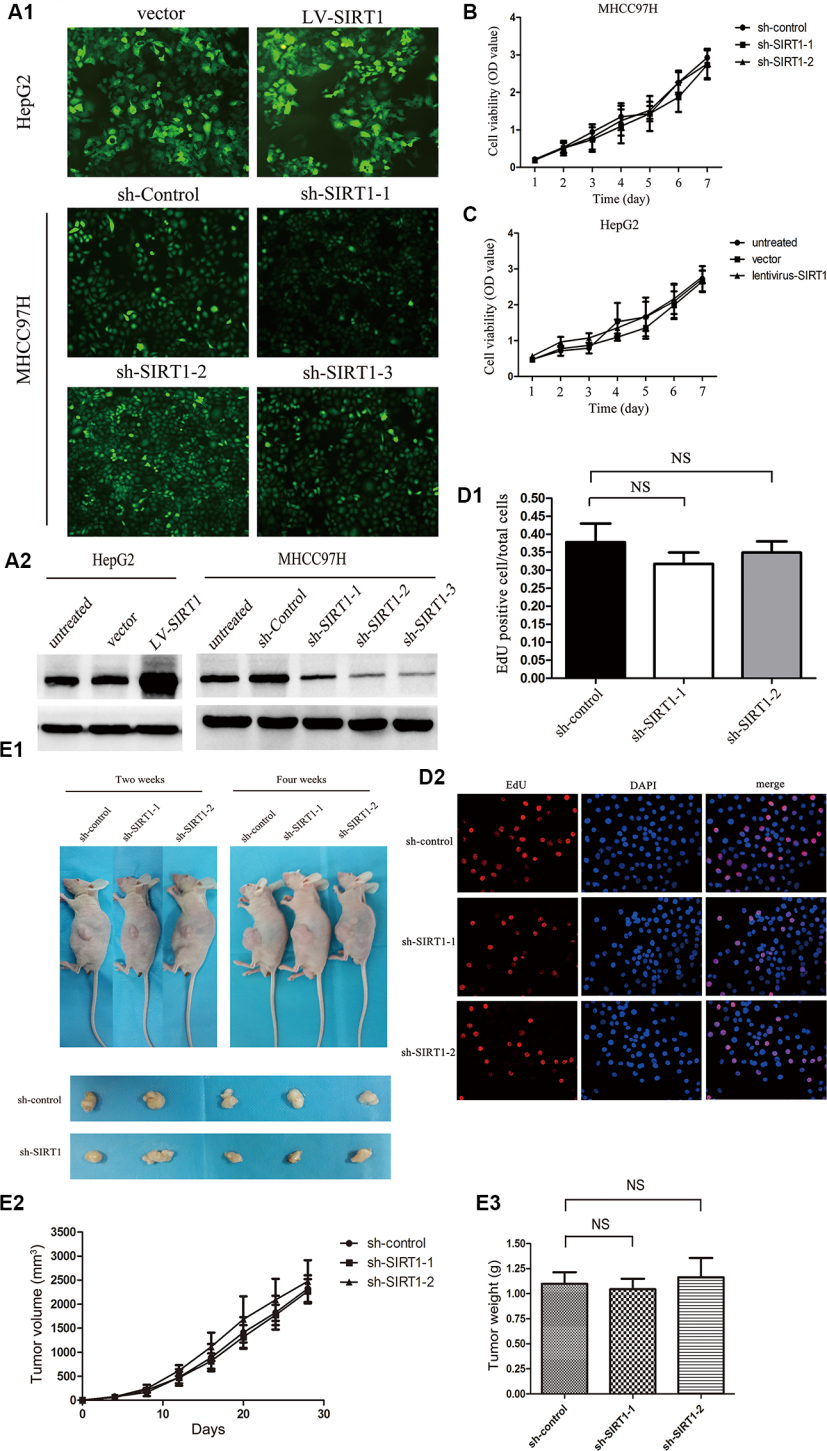
Effect of SIRT1 knockdown on HCC cell proliferation and tumorigenicity (**A1**, **A2**) Representative fluorescent images of stably transfected HepG2-*SIRT1* and MHCC97H-sh-*SIRT1* cells. Western blot analysis confirmed that SIRT1 was overexpressed in HepG2-*SIRT1* cells and effectively downregulated in MHCC97H-sh-*SIRT1* cells. (**B**, **C**) Cell viability was determined each day for seven days by means of a CCK-8 Determination Kit. The OD values are expressed as the mean ± SEM of three independent experiments. (**D1**, **D2**) Representative images of the EdU/DAPI double-stained cells; the percentage of EdU-positive cells was randomly quantified in four different fields from each coverslip. The data were obtained from three independent experiments and expressed as the mean ± SEM. (**E1**, **E2**, **E3**). Representative images of subcutaneous tumors from nude mice that had been inoculated with MHCC97H cells stably expressing control or *SIRT1* shRNA on day 14 and 28. Representative image of the resected subcutaneous tumors after the nude mice had been sacrificed. The tumor volume was measured with calipers every four days (*n* = 4 mice). The tumor weights were measured immediately after the tumors were resected. The data are presented as the mean ± SEM. NS, not significant.

To determine whether SIRT1 is involved in tumor cell proliferation and tumorigenicity in HCC, we established two stable cell lines (denoted HepG2-*SIRT1* and MHCC97H-sh-*SIRT1*) by transfecting cells with the LV-*SIRT1* and LV-sh-*SIRT1* lentiviruses, respectively (Figure 2A1). Both the overexpression and knockdown of SIRT1 were confirmed by Western blotting (Figure 2A2). Three sites were targeted for the knockdown of SIRT1 expression, two of which were effectively downregulated and thus were selected for further study. SIRT1 downregulation and overexpression did not affect the viability of the MHCC97H and HepG2 cells over the course of seven days (Figure 2B, 2C). Cell proliferation was directly assessed by EdU incorporation *in vitro*. As shown in Figure 2D1, 2D2, there was no significant difference in the number of EdU-positive cells between the MHCC97H-sh-*SIRT1* and sh-control transfected cells.

To further investigate the effect of SIRT1 on HCC proliferation *in vivo*, we subcutaneously injected mice with MHCC97H-sh-*SIRT1* cells and dynamically monitored tumor growth (Figure 2E1). Similar tumor growth kinetics and weights were observed in *SIRT1* shRNA-expressing MHCC97H tumors and control shRNA-expressing MHCC97H tumors (Figure 2E2, 2E3). Collectively, these results indicated that SIRT1 expression does not affect HCC proliferation.

### SIRT1 silencing reduced HCC cell invasion and tumor metastasis *in vitro* and *in vivo*

**Figure 3:**
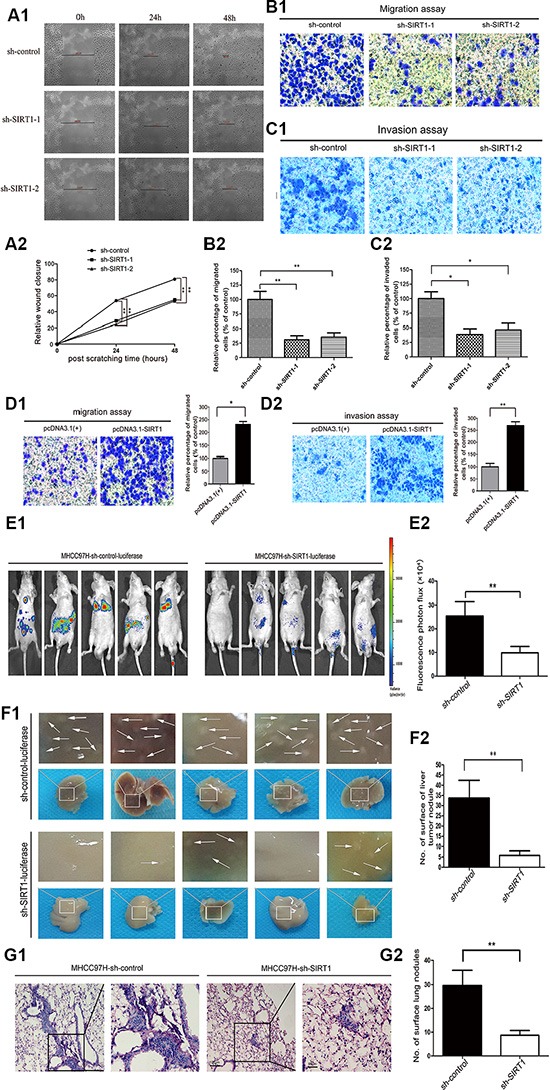
SIRT1 silencing reduces HCC cell invasion and tumor metastasis *in vitro* and *in vivo* (**A1**, **A2**) The wound-healing assay demonstrated that SIRT1 silencing reduced the migration of MHCC97H cells. Representative images were captured at 0, 24, and 48 hours after scratching. The wound closure distance was measured with the software from the Leica Application Suite. (**B1**, **B2**, **C1**, **C2**) Transwell migration/invasion assay showing that SIRT1 depletion reduced the migration and invasion of MHCC97H cells. Representative images of the migratory or invading cells were captured 24 and 36 hours after the cells were inoculated, respectively, and the results are summarized in the lower panel. The results are expressed as the mean ± SEM of three independent experiments (*P < 0.05, **P < 0.01). (**D1**, **D2**) Representative images of the migratory and invading L02 cells were obtained 24 and 36 hours after the cells were inoculated, respectively. (**E1**, **E2**) Representative fluorescent images of nude mice that had been injected through the lateral tail vein with MHCC97H cells stably expressing the vector or sh-*SIRT1*, at four weeks after inoculation (*n* = 5 mice). The fluorescent imaging data are displayed as the photon flux (p/s) and were analyzed with Living Image software. (**F1**, **F2**) Representative images of the tumor nodules on the surface of the liver in the MHCC97H-sh-control group and the MHCC97H-sh-*SIRT1* group. The number of nodules on the surface of the liver was significantly lower in the sh-*SIRT1* group than in the control group, (**P < 0.01). (**G1**, **G2**) Representative images from H&E staining of lung sections from mice that were intravenously injected with MHCC97H-sh-control and MHCC97H-sh-*SIRT1* cells (Original magnification ×100, bar = 100 μm. Designated areas with higher magnification ×200, bar = 50 μm). Significantly fewer nodules were detected on the surfaces of the lungs after SIRT1 knockdown (*n* = 5 mice). The data are expressed as the mean ± SEM (**P < 0.01).

We next investigated the effects of SIRT1 expression on hepatoma cell invasion and metastasis. The wound-healing ability of the stable SIRT1-knockdown MHCC97H cells was significantly attenuated compared to that of the shRNA vector-transfected cells (*P* < 0.01) (Figure 3A1, 3A2). In addition, SIRT1 knockdown markedly reduced the migration (*P* < 0.01) (Figure 3B1, 3B2) and invasion of MHCC97H cells through the Matrigel in the Transwell chamber assay (*P* < 0.05) (Figure 3C1, 3C2). Conversely, *SIRT1* overexpression significantly enhanced the migration and invasion capacities of L02 cells (*P* < 0.05) (Figure 3D1, 3D2). Taken together, these results suggested that SIRT1 increases the motility and invasiveness of HCC cells *in vitro*.

To further determine the function of SIRT1 in tumor metastasis *in vivo*, we constructed stably transfected MHCC97H-sh-control-luciferase and MHCC97H-sh-SIRT1-luciferase cells. Nude mice were intravenously injected with the above cells through the tail vein; representative bioluminescent images of the different groups are shown in Figure 3E1. Mice injected with SIRT1-knockdown cells had dramatically fewer distant organ metastases than those injected with shRNA control cells (Figure 3E2). After six weeks, the mice were sacrificed and the metastatic nodules on the surfaces of the livers and lungs were counted. The tumor nodules on the liver surface were directly counted after the mice were perfused with 0.9% saline, and the tissues were fixed with 4% paraformaldehyde and excised (Figure 3F1). Significantly fewer metastatic nodules were induced on the surfaces of the livers of mice injected with MHCC97H-sh-*SIRT1* cells than of those injected with MHCC97H-sh-control cells (*P* < 0.01) (Figure 3F2). Meanwhile, H&E staining confirmed that the incidence of lung metastasis was significantly lower in the MHCC97H-sh-*SIRT1* group than in the control group (Figure 3G1, 3G2). These data suggested that SIRT1 is required for HCC invasion and metastasis.

### Epithelial-mesenchymal transition was not involved in SIRT1-induced metastasis in HCC cells

**Figure 4:**
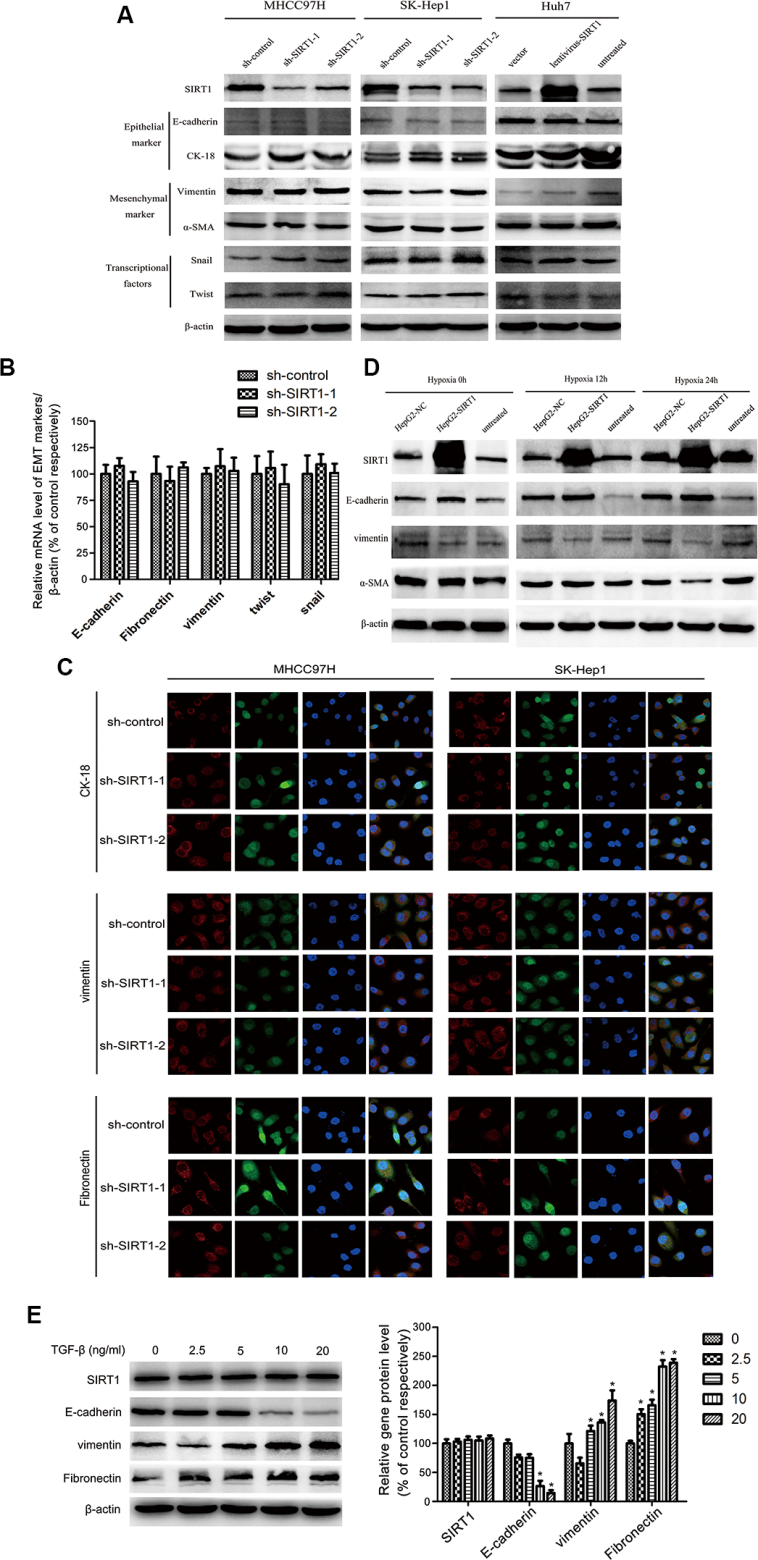
The epithelial-mesenchymal transition (EMT) was not involved in the SIRT1-induced metastasis of HCC cells (**A**) SIRT1 expression did not affect the EMT status in HCC. The Western blot demonstrates that there were no changes in the expression of the epithelial markers E-cadherin and CK-18, the mesenchymal markers vimentin and α-SMA, or the transcriptional factors snail and twist in the MHCC97H-sh-*SIRT1*, SK-Hep1-sh-*SIRT1*, and Huh7-*SIRT1* cells compared to the control cells. (**B**) Real-time PCR analysis of the mRNA levels of key EMT markers (E-cadherin, vimentin, fibronectin, twist and snail) revealed no significant differences between the MHCC97H-sh-*SIRT1* cells and the MHCC97H-sh-control cells. (**C**) Immunofluorescence staining indicated that there was no significant difference in the expression of the EMT markers CK-18, vimentin and fibronectin between SIRT1-depleted MHCC97H or SK-Hep1 cells and the corresponding control cells. (**D**) SIRT1 did not induce the EMT under hypoxic conditions. The expression of the EMT markers E-cadherin, vimentin, and α-SMA did not change in the HepG2-*SIRT1* cells compared to the HepG2-sh-control cells after the cells were cultured in a hypoxic environment (1% O_2_) for 0, 12 or 24 hours. (**E**) SIRT1 was not involved in the TGF-β1-induced EMT. After HepG2 cells were treated with a concentration gradient of TGF-β1 (0, 2.5, 5, 10, 20 ng/mL), Western blot analysis demonstrated that E-cadherin expression was downregulated and vimentin and fibronectin expression were upregulated in a dose-dependent manner, but SIRT1 expression did not change. The relative protein levels are summarized in the right panel (*P < 0.05).

There is abundant evidence of the importance of the EMT in HCC invasion and metastasis [26, 27], and SIRT1 also regulates the EMT program [28]. Therefore, we examined whether the EMT program was activated during SIRT1-induced metastasis. We analyzed the levels of various EMT markers in cells with different SIRT1 levels. After the downregulation of *SIRT1* in MHCC97H and SK-Hep1 (high-EMT) cells and the upregulation of *SIRT1* in Huh7 (low-EMT) cells, the Western blot assay indicated that there were no significant variations in epithelial markers (E-cadherin and CK-18), mesenchymal markers (vimentin and α-SMA) and EMT-related transcription factors (twist and snail) (Figure 4A). In real-time PCR analysis, there were no significant changes in the mRNA levels of key EMT markers (E-cadherin, fibronectin, vimentin, twist, and snail) in the SIRT1-depleted MHCC97H cells compared to the control cells (Figure 4B). Immunofluorescence staining also indicated that SIRT1 silencing did not induce the EMT program in MHCC97H and SK-Hep1 cells (Figure 4C).

Because hypoxia is a vital inducer of the EMT, we further examined whether SIRT1 induces the EMT in hypoxic conditions in HepG2 cells transfected with LV-*SIRT1* and the LV-negative control. The expression of the EMT markers did not change after the cells were cultured in a hypoxic environment (1% O_2_) for 0, 12 or 24 hours (Figure 4D). TGF-β is a canonical inducer of the EMT in HCC cells. Therefore, we induced the EMT in HepG2 cells with different concentrations of TGF-β1 (0, 2.5, 5, 10, and 20 ng/mL). SIRT1 levels did not change under these conditions, whereas E-cadherin was downregulated and fibronectin and vimentin were upregulated (Figure 4E). All of these results suggested that SIRT1 expression does not induce the EMT in HCC cells.

### SIRT1 depletion attenuated mitochondrial biogenesis and biological energy generation in HCC cells

**Figure 5:**
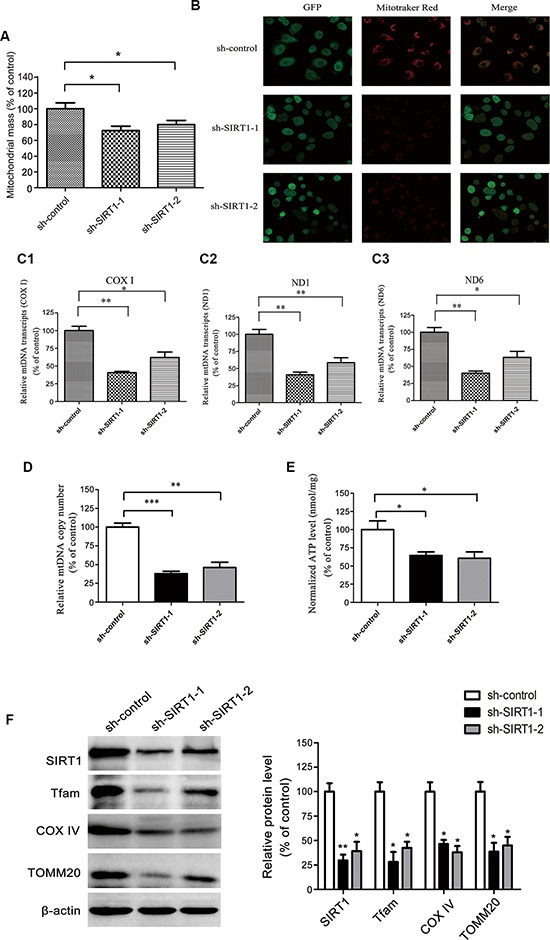
SIRT1 depletion attenuated mitochondrial biogenesis and biological energy production in HCC cells (**A**) NAO staining was used to analyze the mitochondrial mass; the fluorescent intensity was detected with an Infinite^®^ 200 Microplate Reader. (**B**) The MitoTracker^®^ Red CM-H_2_XRos probe was used to further evaluate the mitochondrial mass. After the staining, live cells were visualized with a Leica confocal laser scanning microscope, original magnification ×100. (**C1**, **C2**, **C3**) Real-time PCR was used to measure the levels of the mitochondrial genes *COXI*, *ND1*, and *ND6* as representative mtDNA transcripts. The relative mtDNA transcript levels were normalized to the internal control, β-actin. (**D**) Quantitative real-time PCR analysis was used to detect the mtDNA copy number. (**E**) ATP concentrations were determined with an ATP Determination Kit. (**F**) Western blot analysis of the expression of the mitochondrial biogenesis-related proteins TFAM, COXIV and TOMM20 in SIRT1-depleted MHCC97H cells compared to sh-control cells. The densitometric analysis of these proteins is summarized in the right panel. The data are expressed as the mean ± SEM of three independent experiments. These results are expressed as a percentage of control, which was set to 100%. *P < 0.05, **P < 0.01, ***P < 0.001 versus the control group.

Mitochondrial biogenesis increases energetic metabolism and thus facilitates the motility of tumor cells. In our study, the stable SIRT1-knockdown and shRNA vector-expressing MHCC97H cells were stained with the mitochondria-specific dye NAO, and 28% and 20% reductions in the mitochondrial mass, respectively, were observed after SIRT1 was downregulated (Figure 5A). The reduction in mitochondrial mass was also confirmed by the reduced MitoTracker^®^ Red CM-H_2_XRos staining in the sh-*SIRT1*-MHCC97H cells (Figure 5B).

Impaired mitochondrial biogenesis, *COX I*, *ND1* and *ND6* as representative mtDNA transcripts. The levels of these mtDNA transcripts were lower in the sh-*SIRT1*-MHCC97H cells than in the shRNA vector-expressing cells (Figure 5C1–5C3). SIRT1 knockdown in the MHCC97H cells reduced the mtDNA copy number to 38% and 46% of that in the control cells, respectively (Figure 5D).

In mitochondrial functional analysis, intracellular ATP levels in SIRT1-depleted MHCC97H cells were reduced by 36% and 39%, respectively, compared to the control cells (Figure 5E). Mitochondrial transcription factor A (TFAM) is a key regulator of mitochondrial transcription initiation and the mtDNA copy number replication [29]. Thus, we determined the levels of TFAM and other mitochondrial biogenesis-related proteins (COXIV and TOMM20). SIRT1 knockdown in MHCC97H cells reduced the expression of the TFAM, COXIV and TOMM20 proteins (Figure 5F). Therefore, SIRT1 depletion in HCC cells reduced the capacity for mitochondrial biogenesis and impaired mitochondrial function, leading to deficient bioenergetic production.

### PGC-1α overexpression enhanced mitochondrial biogenesis and was responsible for SIRT1-facilitated HCC metastasis

**Figure 6:**
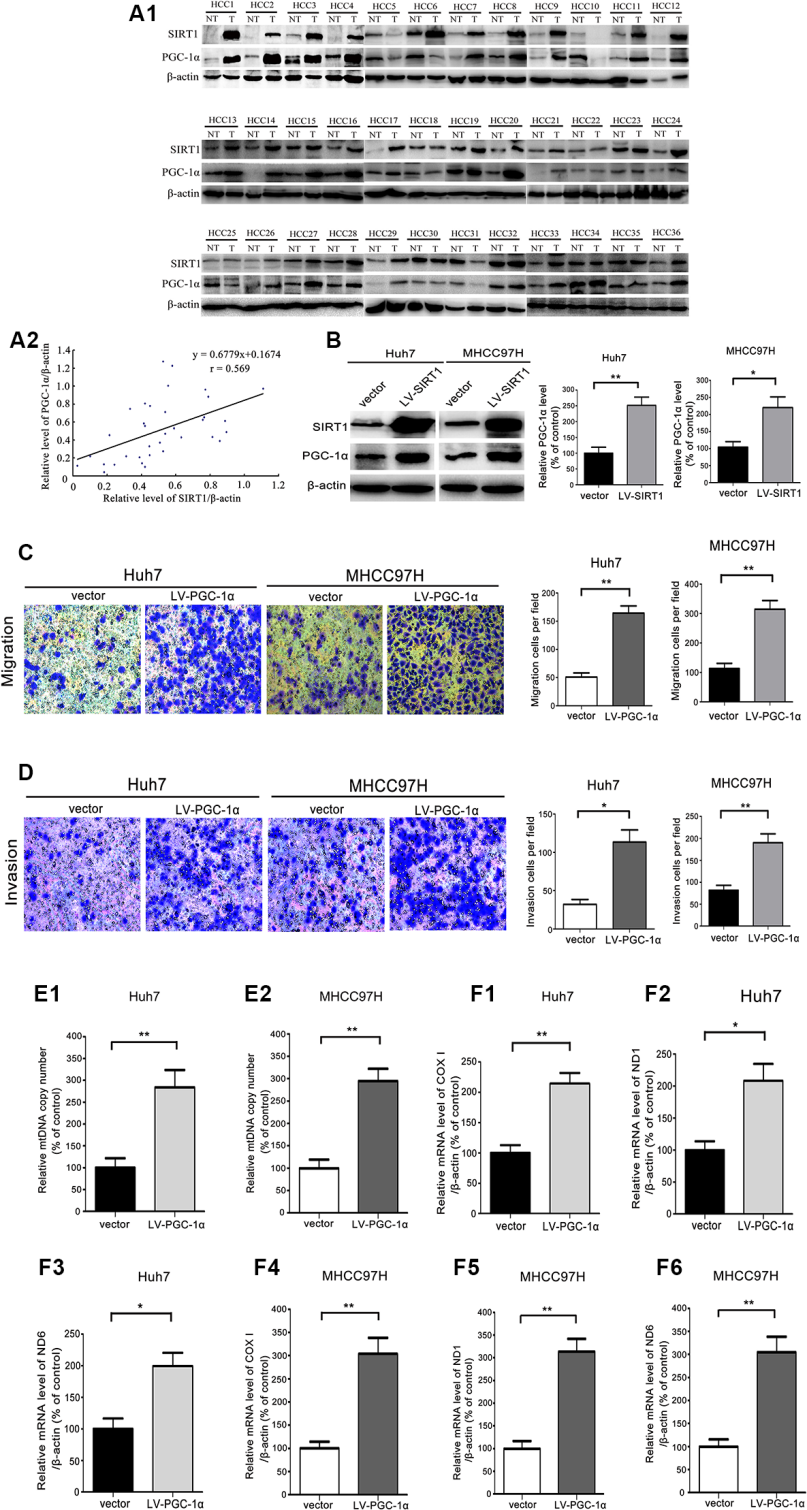
PGC-1α promoted HCC cells migration and invasion by enhancing mitochondrial biogenesis (**A1**, **A2**) Western blot analysis of SIRT1 and PGC-1α expression in the HCC tumor specimens. Correlation analysis revealed a significant association between SIRT1 overexpression and the PGC-1α level in HCC specimens (*r* = 0.569, *P* < 0.001). β-actin was used as the internal standard for equal protein loading, NT: adjacent nontumoral liver; T: HCC tumor tissue. (**B**) A representative immunoblot and quantification analysis of the PGC-1α levels in MHCC97H and Huh7 cells transfected with lentivirus (LV)-*SIRT1* and the corresponding control cells. β-actin was used as the internal standard for equal protein loading. (**C**) Representative images and quantification analysis of the migratory Huh7 and MHCC97H cells that had been stably transfected with the vector or LV-*SIRT1*. The images were captured 24 hours after the cells were inoculated. (**D**) Representative images and quantification analysis of the invading Huh7 and MHCC97H cells that stably expressed the vector or LV-*SIRT1*. The images were captured 36 hours after the cells were inoculated. (**E**) The mtDNA copy number and (**F1**–**F6**) the levels of the mitochondrial genes *COXI*, *ND1*, and *ND6* were analyzed by quantitative real-time PCR. The data are representative of three independent experiments. The values are presented as the mean ± SEM. *P < 0.05, **P <0.01, versus the control group.

PGC-1α is a master integrator of the cellular signals that regulate mitochondrial biogenesis and oxidative phosphorylation [20, 30]. First, in our study, we detected SIRT1 and PGC-1α levels in HCC samples simutaneously, and observed that SIRT1 overexpression was highly associated with the upregulation of PGC-1α in HCC tumors compared to adjacent nontumoral liver tissues (*r* = 0.569, *P* < 0.001) (Figure 6A1, 6A2). Moreover, forced expression of *SIRT1* in Huh7 and MHCC97H cells significantly increased PGC-1α expression (Figure 6B), which further demonstrated that SIRT1 positively regulates PGC-1α expression. Therefore, we also explored the relationship between PGC-1α expression and HCC metastasis. Ectopic expression of *PGC-1α* in Huh7 and MHCC97H cells significantly increased cellular migration (Figure 6C) and invasion (Figure 6D). In addition, ectopic expression of *PGC-1α* enhanced the mtDNA copy number by 2.7-fold and 2.9-fold in Huh7 and MHCC97H cells, respectively, compared to control cells (Figure 6E1, 6E2). The levels of the mtDNA transcripts for*COXI*, *ND1* and *ND6* were significantly greater in LV-*PGC-1α*-transfected Huh7 and MHCC97H cells than in control cells (Figure 6F1–6F6). Thus, we can conclude that PGC-1α promotes HCC cell migration and invasion by enhancing mitochondrial biogenesis.

**Figure 7:**
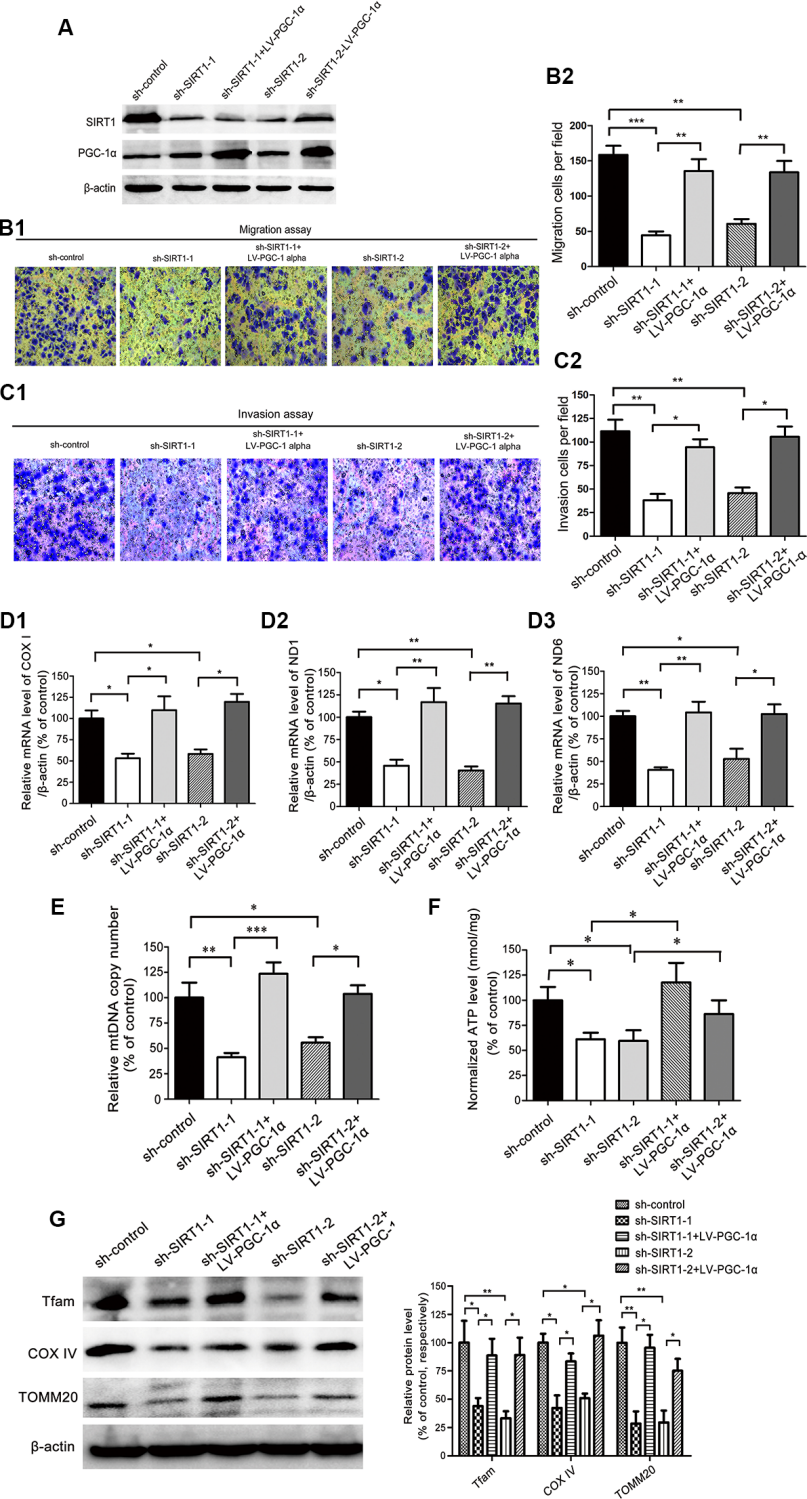
PGC-1α overexpression-induced mitochondrial biogenesis was responsible for SIRT1-facilitated HCC metastasis (**A**) Establishment of the stably transfected MHCC97H- sh-*SIRT1*-LV-*PGC-1α* cells. Western blotting was used to verify the transfection efficiency. β-actin was used as the internal standard for equal protein loading. (**B**, **C**) The Transwell migration and invasion assay demonstrated that PGC-1α overexpression restored the migration and invasion of the SIRT1-knockdown MHCC97H cells. Representative images and quantification of the migratory and invading cells were obtained 24 and 36 hours after the cells were inoculated, respectively. (**D1**, **D2**, **D3**) The mitochondrial genes *COXI*, *ND1*, *ND6* and (**E**) the mtDNA copy number were analyzed by quantitative real-time PCR. The relative levels of the mtDNA transcripts and the mtDNA copy number were normalized to the internal control, β-actin. (**F**) ATP concentrations were determined with an ATP Determination Kit. (**G**) Representative immunoblots and quantification of the levels of the mitochondrial biogenesis-related proteins TFAM, COXIV, and TOMM20 after *PGC-1α* was overexpressed in SIRT1-depleted MHCC97H cells. β-actin was used as the internal standard for equal protein loading. These data are expressed as the mean ± SEM from three independent experiments. *P < 0.05, **P <0.01, ***P < 0.001 versus the control group.

LV-*PGC-1α* was transfected into SIRT1-depleted MHCC97H cells as a further test of whether SIRT1 facilitates HCC metastasis via a PGC-1α-induced increase in mitochondrial biogenesis (Figure 7A). Importantly, forced expression of *PGC-1α* significantly restored the cell migration (Figure 7B1, 7B2) and enhanced the cellular invasion (Figure 7C1, 7C2) that were inhibited by SIRT1 depletion. In real-time PCR assays, the mRNA levels of *COXI*, *ND1* and *ND6* were significantly greater and the mtDNA copy number was clearly enhanced after*PGC-1α* was overexpressed in SIRT1-depleted MHCC97H cells (Figure 7D1–7D3, 7E). Ectopic expression of *PGC-1α* increased intracellular ATP levels by 56% and 32% compared to SIRT1-depleted MHCC97H cells (Figure 7F). The expression of the mitochondrial biogenesis-related proteins TFAM, COXIV and TOMM20 significantly increased in SIRT1-depleted MHCC97H cells following transfection with LV-*PGC-1α* (Figure 7G). Collectively, these data suggested that *PGC-1α* overexpression reversed the inhibitory effects of SIRT1 knockdown on invasion and mitochondrial biogenesis.

### PGC-1α reversed the suppressive effects of SIRT1 depletion on metastasis by enhancing mitochondrial biogenesis *in vivo*

**Figure 8:**
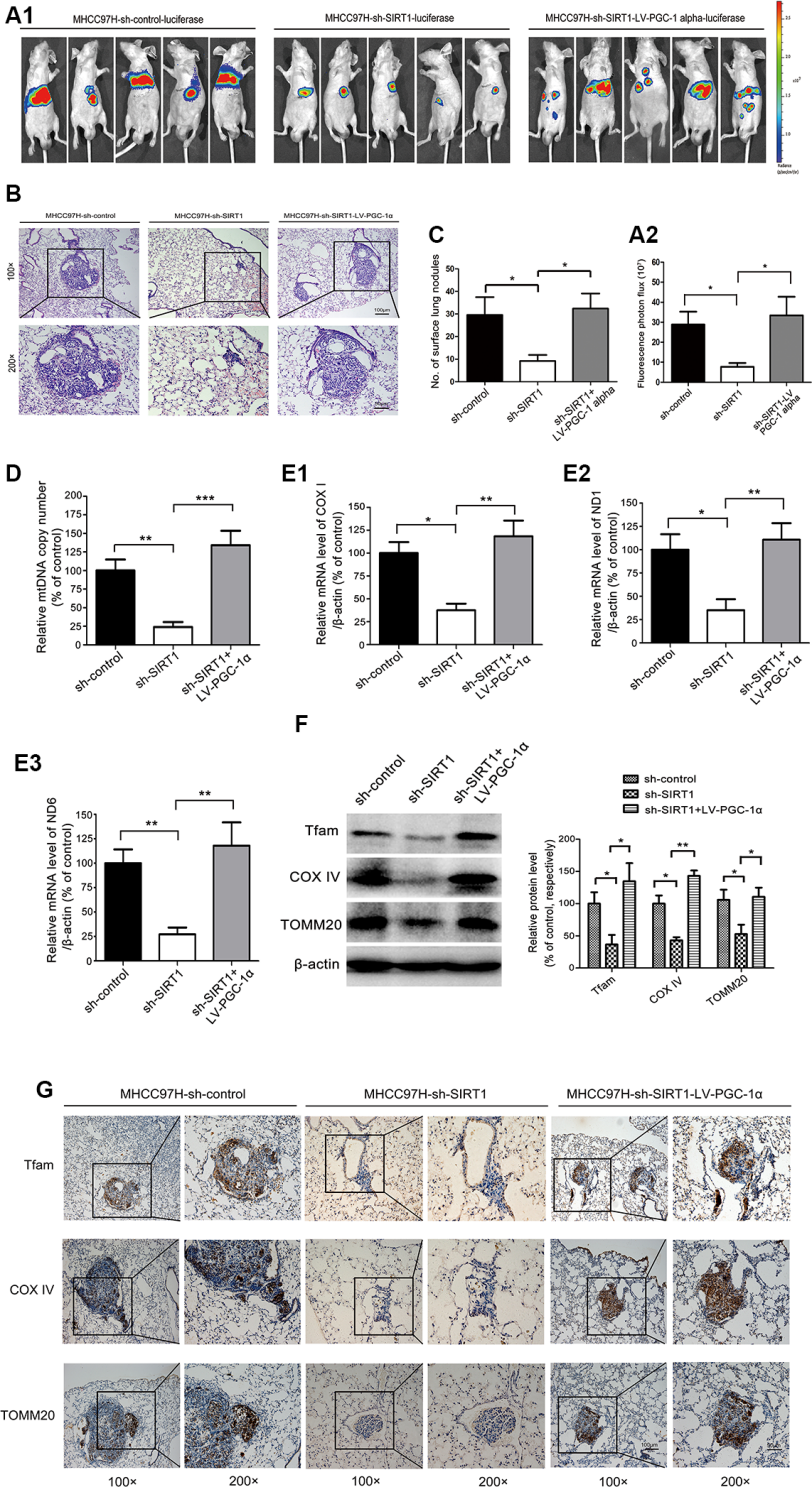
PGC-1α reversed the inhibitory effects of SIRT1 depletion on metastasis by enhancing mitochondrial biogenesis *in vivo* (**A1**, **A2**) Representative fluorescent images of nude mice that had been inoculated with the MHCC97H-vector, MHCC97H-sh-*SIRT1* or MHCC97H-sh-*SIRT1*-LV-*PGC-1α* cells in the liver parenchyma; these cells expressed firefly luciferase (blue, least intense; red, most intense) (*n* = 5). The fluorescent imaging data were analyzed with the Bioluminescence Imaging software. (**B**, **C**) Representative images of the H&E-stained lung sections of mice harbouring the orthotopic MHCC97H-vector, MHCC97H-sh-*SIRT1* or MHCC97H-sh-*SIRT1*-LV-*PGC-1α* cells; the number of tumor nodules in the lungs was determined. The data are presented as the mean ± SEM, *P < 0.05 versus the control group. Quantitative real-time PCR of (**D**) the mtDNA copy number and (**E1**–**E3**) the mitochondrial genes *COXI*, *ND1*, and *ND6* (representative mtDNA transcripts) from the DNA and mRNA extracted from the lung tissue of nude mice; β-actin was used as the internal control. These results are expressed as a percentage of the control group, which was set to 100%. The data are presented as the mean ± SEM, **P < 0.01 versus the control group. (**F**) Representative immunoblots and quantification of the levels of the mitochondrial biogenesis-related proteins TFAM, COXIV and TOMM20; total protein was extracted from the lung tissue of nude mice; β-actin was used as the internal standard for equal protein loading. The data are presented as the mean ± SEM, *P < 0.05, **P < 0.01 versus the control group. (**G**) Representative immunohistochemical images of the expression of the mitochondrial biogenesis-related proteins TFAM, COXIV, and TOMM20 in the metastatic loci of lung tissues from nude mice (Original magnification ×100, bar = 100 μm. Designated areas with higher magnification ×200, bar = 50 μm).

To demonstrate the interaction of PGC-1α with SIRT1 *in vivo*, we established an orthotopic transplantation model in nude mice to simulate cell growth, dissemination and metastasis (Figure 8A1). First, stably transfected HCC cell lines (MHCC97H-sh-control-*Luc*, MHCC97H-sh-*SIRT1*-*Luc*, MHCC97H-sh-*SIRT1*-LV-*PGC-1α*-*Luc*) were established to achieve the lentivirus-mediated knockdown of SIRT1 and overexpression of *PGC-1α*. In Bioluminescence Imaging Analysis, the fluorescence photon flux (which has a linear relationship with the number of tumor cells) was significantly reduced in SIRT1-depleted cells compared to control cells, but this inhibitory effect could be reversed by the overexpression of *PGC-1α* (Figure 8A2). Smaller metastatic foci were prevalent in the MHCC97H-sh-*SIRT1* group, but the size and number of lung tumor nodules was obviously greater in the *PGC-1α* overexpression (MHCC97H-sh-*SIRT1*-LV-*PGC-1α*) group (Figure 8B). The number of lung tumor nodules in the MHCC97H-sh-*SIRT1* group was significantly lower than that in the MHCC97H-sh-control group, but the number in the MHCC97H-sh-*SIRT1*-LV-*PGC-1α* group was significantly higher than that in the MHCC97H-sh-*SIRT1* group and nearly equal to that in the MHCC97H-sh-control group (Figure 8C).

We also extracted the DNA and mRNA from the lung tissues of the nude mice, and found that the mtDNA copy number and the levels of mtDNA transcripts (*COXI*, *ND1* and *ND6*) were significantly lower in the MHCC97H-sh-*SIRT1* group than in the MHCC97H-sh-control group, and *PGC-1α* overexpression restored these levels (Figure 8D, 8E1–8E3). In addition, Western blot analysis of the lung tissue revealed that the levels of the mitochondrial biogenesis-related proteins TFAM, COXIV and TOMM20 were significantly lower in MHCC97H-sh-*SIRT1* group than in the MHCC97H-sh-control group, while *PGC-1α* overexpression enhanced the expression of these genes (Figure 8F). Likewise, immunohistochemical analysis directly demonstrated that the level of TFAM, COXIV, and TOMM20 were significantly lower in the MHCC97H-sh-*SIRT1* group than in the MHCC97H-sh-control group, while ectopic expression of *PGC-1α* distinctly increased these levels (Figure 8G). Taken together, these results strongly suggested that a PGC-1α-mediated increase in mitochondrial biogenesis is critical for promoting SIRT1-overexpression- facilitated HCC metastasis.

## DISCUSSION

SIRT1 may act as a tumor promoter or suppressor, depending on the tumor type, as its overexpression inhibits tumorigenesis in colon tumors and ovarian cancer [31, 32], but stimulates tumorigenesis in prostate tumors and acute myeloid leukemia [11, 33]. In this study, we demonstrated that SIRT1 was frequently upregulated in human HCC tissues (overexpression rate: 56.9%), compared to adjacent nontumoral liver tissues. SIRT1 overexpression correlated with portal vein tumor thrombus formation and advanced TNM stages, but was not associated with other clinical parameters (Table 1), indicating that SIRT1 expression is associated with hematogenous metastasis in HCC. Additionally, HCC patients with high levels of SIRT1 had shorter disease-free survival times and lower overall survival rates than those with low levels of SIRT1. Taken together, these data strongly suggested that SIRT1 contributes to HCC progression and may serve as a useful prognostic biomarker.

Although SIRT1 stimulates cancer proliferation by blocking cellular senescence and disrupting cell-cycle arrest [8, 34], Wang considered SIRT1 to be a tumor suppressor, given that the activation of SIRT1 by resveratrol partially inhibited tumor formation [35]. In our study, SIRT1 expression had no apparent effects on cell growth and proliferation *in vitro*, and did not affect xenograft formation *in vivo*. Our results were consistent with those of Lee and colleagues, who found that colony formation (which reflects cell growth) was identical in HepG2 cells transfected with wild-type *SIRT1* or the T344A mutant compared to the HepG2 cells transfected with vector [36]. The significant correlation between SIRT1 expression and microvascular invasion and advanced TNM stages prompted us to investigate the role of SIRT1 in HCC cell migration and metastasis. SIRT1 knockdown significantly reduced the migration and invasion of HCC cells *in vitro*, and reduced the formation of metastatic foci *in vivo*, while the ectopic expression of SIRT1 in immortal liver cells enhanced their invasiveness. All of these results suggested that SIRT1 expression promotes HCC progression by facilitating invasion and metastasis.

Our previous study demonstrated that the EMT was involved in the dissemination of HCC cells from the site of the primary tumor [37]. Moreover, SIRT1 has been found to promote prostate cancer metastasis by inducing EMT, but to prevent lung cancer metastasis by blocking the EMT [11, 38]. Therefore, it was necessary to determine whether SIRT1 activates the EMT in HCC. In the present study, multiple assays demostrated that the EMT program was not activated after SIRT1 knockdown or upregulation in HCC cells. Hypoxia has been reported to initiate the EMT program through a number of key transcriptional factors [39]. We found that hypoxia induced the EMT, but not through a SIRT1-dependent pathway. We established an additional EMT model by treating the HCC cells with TGF-β1, a major inducer of the EMT [40], and found that SIRT1 did not participate in the EMT process. Taken together, SIRT1 expression facilitated HCC invasion and metastasis but did not activate the EMT program.

Breast cancer cells display metabolic heterogeneity and their distinct metabolic reprogramming defines their sites of metastasis [41]. Increased cancer invasiveness is associated with higher mitochondrial activity, elevated ATP production, oxygen consumption and pyruvate uptake [42]. Moreover, treatment with a mitochondrial uncoupling agent or ATP synthesis inhibitor reduced lamellipodia formation and decreased breast cancer cell migration and invasion [43]. Therefore, it is necessary to investigate whether mitochondrial biogenesis was involved in the SIRT1 knockdown-mediated reduction of HCC invasion. In accord with our hypothesis, SIRT1 knockdown decreased the mitochondrial mass, mtDNA copy number, mtDNA transcript levels and the levels of mitochondrial biogenesis-related proteins in HCC cells. More importantly, the intracellular ATP production was obviously decreased in the SIRT1-depleted cells. Taken together, these results suggested that SIRT1 depletion decreased the invasion and metastasis by attenuating mitochondrial biogenesis and energy production in HCC cells.

The energy levels of the tumorigenic metabolism are maintained by increased mitochondrial biogenesis and controlled by PGC-1α [44]. Resveratrol increased the PGC-1α levels and its activity by activating SIRT1 in muscle cells [45]. Therefore, it is extremely necessary to determine the relationship between SIRT1 expression and PGC-1α in HCC. In our study, the significant correlation between SIRT1 overexpression and PGC-1α levels in the HCC specimens reminded us that SIRT1 may regulate PGC-1α expression. In accord with our hypothesis, the ectopic expression of SIRT1 significantly upregulated the levels of PGC-1α. Subsequently, our study provided the first evidence that the ectopic expression of PGC-1α enhanced the migration and invasion of HCC cells. The increased mtDNA copy numbers and transcript levels indicated that PGC-1α significantly increased mitochondrial biogenesis. Therefore, we can conclude that PGC-1α expression may promote the migration and invasion of HCC cells by increasing mitochondrial biogenesis.

Recently, considerable attention has been focused on the effects of the PGC-1α-mediated pathway on tumorigenesis, as high PGC-1α levels in melanoma cells increased the mitochondrial capacity to resist oxidative stress [46]. As mentioned above, PGC-1α promoted the formation of distant metastasis by enhancing mitochondrial biogenesis in invasive breast cancer [16]. Nevertheless, it remained unclear whether the PGC-1α-mediated increase in mitochondrial biogenesis participated in SIRT1-facilitated HCC metastasis. It was reassuring that PGC-1α overexpression restored the migration and invasion of HCC cells that had been reduced by SIRT1 knockdown. Moreover, the ectopic expression of PGC-1α in SIRT1-depleted MHCC97H cells restored mitochondrial biogenesis and ATP production, which further confirmed that SIRT1 facilitates HCC invasion by activating the SIRT1/PGC-1α axis to increase mitochondrial biogenesis. Subsequently, we established a metastatic orthotopic transplantation model to demonstrated that SIRT1 interacts with PGC-1α *in vivo*. In line with our expectations, Bioluminescence Imaging revealed that PGC-1α overexpression abolished the inhibitory effect of SIRT1 knockdown on HCC cell metastasis, and even increased the number of metastatic loci. What's more, the mtDNA copy numbers and transcript levels, as well as the levels of the mitochondrial biogenesis-related proteins TFAM, COXIV and TOMM20 in metastatic lung tissues, were significantly lower in the SIRT1-depleted group than in the shRNA-control group, and their levels were restored by the overexpression of *PGC-1α*. It is noteworthy that TFAM is a critical downstream PGC-1α mediator and stimulates mtDNA replication and mitochondrial gene expression, which are essential for mitochondrial biogenesis [47]. Taken together, these findings suggested that SIRT1 depletion inhibited mitochondrial biogenesis and thus impaired metastasis in HCC, but these effects could be reversed by ectopic *PGC-1α* expression.

In summary, SIRT1 overexpression in HCC correlated with microvascular invasion and advanced TNM stages and predicted poor outcomes, which confirmed that the SIRT1 level could be a promising clinical prognostic biomarker. Ectopic expression of *PGC-1α* reversed the inhibitory effects of SIRT1 depletion on the invasion and metastasis of HCC cells, by enhancing mitochondrial biogenesis and ATP production *in vitro* and *in vivo*. Additional investigations are necessary to determine how SIRT1 binds to PGC-1α and regulates its activity; such studies may uncover an effective therapeutic target in the SIRT1/PGC-1α axis and ultimately improve the prognosis of HCC patients.

## MATERIALS AND METHODS

### Cell culture

SK-Hep1, PLC and SMMC-7721 cells were purchased from the Chinese Academy of Sciences Type Culture Collection Cell Bank. HepG2 cells were obtained from the American Type Culture Collection (Manassas, VA). LO2, Hep3B and Huh7 cells were a kind gift from Dr. Ni Zhenghong (Department of Biochemistry at the Third Military Medical University, Chongqing, China). The MHCC97H cell line was acquired from the China Center for Type Culture Collection.

The human hepatoma cell lines (HepG2, PLC, SMMC-7721, Hep3B and Huh7) were cultured in Dulbecco's modified Eagle's medium (Gibco, Invitrogen, Carlsbad, CA) supplemented with 10% FBS (Gibco BRL, Grand Island, NY). The SK-Hep1 cells were cultured in Minimum Essential Medium (Hyclone Logan, UT) supplemented with 15% FBS (Gibco BRL). The immortalized liver cell line (L02) and the hepatoma cell line (MHCC97H) were cultured in RPMI 1640 (Gibco Life Technologies, Grand Island, NY) supplemented with 10% FBS (Gibco BRL). All cells were maintained at 37°C in a humidified atmosphere containing 5% CO_2_, and were harvested with trypsin before use.

### HCC patients and samples

The liver tumor tissues and corresponding adjacent nontumoral liver tissues were immediately collected from 72 HCC patients during curative hepatectomy at The Xin Qiao Hospital of the Third Military Medical University (Chongqing, China). The samples were obtained immediately after resection, snap-frozen and stored at −80°C until further investigation. The paraffin-embedded samples were freshly cut into 5-μm sections and mounted on microscope slides before immunohistochemistry. All specimens were diagnosed by histopathological examination. Clinical and pathological features were retrieved and follow-up information was obtained at regular intervals. The clinical characteristics of the HCC patients are summarized in Table 1. Written informed consent was obtained from each patient, and the study protocol was approved by the Ethics and Scientific Committees of our institution.

### Plasmid constructs and lentivirus production

Full-length human *SIRT1* was synthesized and cloned into the pcDNA3.1(+) expression vector by Sangon Biotech (Shanghai, China) and then transfected into L02 cells with Lipofectamine 2000 (Invitrogen) according to the manufacturer's instructions. Cells transfected with an empty vector were used as controls. A lentivirus-*SIRT1* vector system (pLV-EF1a-GFP/puro) was constructed, packaged, and purified by GeneChem (Shanghai, China). Lentiviral constructs expressing *SIRT1* shRNA (pGCSIL-GFP) were purchased from GeneChem (Shanghai, China). The *SIRT1* shRNA targeting sequence was as follows: 5ʹ- GGGAATCCAAAGGATAATT-3ʹ, 5ʹ-GGCTTGATGGTAATCAGTA-3ʹ and 5ʹ- AAAGCCTT TCTGAATCTAT-3ʹ. A lentivirus-*PGC-1α* vector system (Ubi-MCS-3FLAG- SV40-EGFP-IRES-puromycin) was purchased from GeneChem (Shanghai, China). A lentivirus-sh-SIRT1 vector system containing firefly luciferase (hU6-MCS-Ubiquitin-*Luc_firefly*-IRES-puromycin) was constructed, packaged, and purified by GeneChem, and was used to study metastasis in animals via bioluminescence imaging.

### Establishment of stably transfected cells

Stable transfectants of SIRT1-knockdown MHCC97H cells (high metastatic potential), *SIRT1*-overexpressing HepG2 and Huh7 cells (low metastatic potential) and *PGC-1α*- overexpressing MHCC97H-sh-*SIRT1* cells were constructed according to the manufacturer's protocols. Briefly, the cells were seeded onto six-well plates at a concentration of 0.5 × 10^5^ cells/well on the day before lentivirus transfection. *SIRT1*-shRNA-LV, *SIRT1*-overexpression-LV and *PGC-1α*-LV were transfected into cells at a multiplicity of infection (MOI) of 10 (MHCC97H and Huh7) or 20 (HepG2) by means of polybrene (6 μg/mL) (Santa Cruz Biotechnology, Inc, CA) and the Enhanced Infection Solution from Gene Chem. Meanwhile, a negative control lentivirus GFP-LV (Gene Chem) was transfected into the same cells. Seventy-two hours after transfection, the transfection effects were observed with a fluorescence microscope (Leica TCS SP5 MP, wetzlar, Germany). Subsequently, Puromycin (final concentration: 5 μg/mL) (Santa Cruz Biotechnology, Inc.) was used to select stable clones for the establishment of stably transfected cell lines.

### Cell viability and DNA synthesis assay

Cell viability was detected with the Cell Counting Kit-8 (CCK-8, Dojindo, Kumamoto, Japan) according to the manufacturer's instructions. Briefly, 0.2 × 10^4^ stably transfected cells were seeded onto 96-well plates, mixed with 10 μL of CCK-8 solution per well and incubated for two hours at 37°C. Optical density (OD) values were determined in each well at an absorbance of 450 nm on an Infinite^®^ 200 Microplate Reader (Tecan, Mannedorf, Switzerland). Proliferation was determined based on the amount of DNA synthesis in the cells, which was measured with a Cell-Light^™^ EdU Apollo 567 *In vitro* Kit (RIBOBIO, Guangzhou, China) according to the manufacturer's instructions. Briefly, 5 × 10^4^ stable SIRT1-knockout cells were seeded onto poly-L-lysine-coated coverslips in 24-well plates. Once the cells had reached the logarithmic growth phase, they were incubated with EdU medium (50 μM) for two hours at 37°C. Then, the cells were fixed with 4% paraformaldehyde in PBS (pH 7.4) for 30 minutes at room temperature. After the cells were rinsed twice with PBS, they were stained with 1× Apollo staining solution for 30 minutes. After staining, the cells were washed three times with PBS containing 0.25% Triton X-100. The nuclei were counterstained with 4,6-diamidino-2-phenylindole (DAPI). The EdU-positive and total cell numbers were counted on each coverslip in four different non-overlapping fields under a fluorescence microscope (Leica, Germany).

### *In vitro* migration, invasion and wound-healing assays

Cell motility was assessed with the Transwell migration assay and the wound-healing assay. Cell invasion was analyzed with the Matrigel invasion assay. The Transwell migration assay was performed in 12-well plates using Transwell cell culture inserts with an 8-μm pore size PET membrane (Millipore, Billerica, MA). Briefly, for the Transwell migration and invasion assays, 1 × 10^5^ cells and 2 × 10^5^ cells, respectively, were plated in the top chamber. For the invasion assay, the chamber inserts were coated with 200 mg/mL of Corning Matrigel matrix and dried for two hours in a 37°C incubator before the cells were inoculated. The cells that had migrated to the underside of the membrane were fixed, stained with crystal Violet, and subsequently counted in 10 microscopic fields. The mean value was expressed as a percentage relative to the negative control. Both experiments were independently repeated in triplicate. For the wound-healing assay, the stable SIRT1-knockdown and vector-transfected MHCC97H cells were seeded onto six-well plates. The cells were grown to confluency and gently scratched with a sterile 200 μL pipette tip to create a parallel wound. The images of the migrating cells were captured at 0, 24, and 48 hours after scratching. Then, the distance between the wound edges was calculated with software from the Leica Application Suite (Leica, Germany) and the experiments were independently performed in triplicate.

### Mitochondrial mass determination

The mitochondrial mass was determined by staining with 10-N-nonyl-acridine orange (NAO) (Invitrogen, A1372) and MitoTracker^®^ Red CM-H_2_XRos (Invitrogen, M7513) according to the manufacturer's instructions. For NAO staining, the stably transfected cells were seeded at a final concentration of 0.5 × 10^4^ cells/well in a 96-well plate. The next day, the cells were treated with 5-μM NAO for 30 minutes. After the cells were washed twice with PBS, an Infinite^®^ 200 Microplate Reader was used to measure the emitted fluorescence at 530 nm following excitation at 485 nm. The MitoTracker^®^ Red CM-H_2_XRos probe was used to further investigate the changes in mitochondrial mass. Briefly, 1 × 10^5^ stably transfected cells were seeded onto cell culture dishes (20 mm). When the cells reached 80% confluency, the medium was removed and a pre-warmed (37°C) staining solution containing 100 nM MitoTracker^®^ Red CM-H_2_XRos was added. After incubation for 20 minutes at 37°C and washes with PBS, the live cells were visualized on a Leica confocal laser scanning microscope (Leica TCS SP5 MP, Wetzlar, Germany). Both experiments were independently repeated in triplicate.

### Relative mitochondrial DNA content measurements

Mitochondrial DNA (mtDNA) was measured by real-time PCR analysis of the total DNA extracted from cells and mouse tissues. Total cellular and tissue DNA were extracted with an E.Z.N.A^™.^ Tissue DNA Kit (Omega, Atlanta, GA) according to the manufacturer's instructions. For mtDNA content analysis, the mtDNA copy number was assessed with a specific probe for Complex II (succinate-ubiquinone oxidoreductase) mtDNA (Human Complex II: 5ʹ-CAAACCTACGCCAAAATCCA-3ʹ and 5ʹ-GAAATGAATGAGCCTACAG A-3ʹ) [48]. In addition, mtDNA transcript levels were determined for a series of mitochondrially encoded genes, including cytochrome c oxidase subunit I (*COXI*) encoded by the heavy chain of mtDNA, NADH dehydrogenase subunit 6 (*ND6*) encoded by the light chain of mtDNA, and NADH dehydrogenase subunit 1 (*ND1*) [49]. The mtDNA primers for *COXI* were 5ʹ-CCACTTCGCCATCATATTCGTAG G-3ʹ and 5ʹ-TCTGAGTAGCGTCGTG GTATTCC-3ʹ. The primers for *ND6* were 5ʹ-TCACCCAGCTACCACCATCATTC-3ʹ and 5ʹ-CACTGAGGAGTACCCAGAGACTTG-3ʹ. The primers for *ND1* were 5ʹ-TCCTAACA CTCCTCGTCCCTA TTC-3ʹ and 5ʹ-GGATGCCGTATGGACCTACAATG-3ʹ. β-actin was used as the internal standard. The primers for β-actin were 5ʹ-CCACACCCGCCACCAGT TC-3ʹ and 5ʹ-CCTTCTGACCCATTCCCACCATC-3ʹ. The cycle threshold (Ct) values for β-actin, the mtDNA copy number, and the mtDNA transcripts were determined in each individual quantitative PCR run. The ΔΔCt method (mtDNA copy number/transcripts relative to β-actin) was used to quantify the mtDNA transcripts in a cell. Each measurement was repeated five times and normalized against the control.

### ATP measurements

ATP levels were measured with an ATP Determination Kit (Beyotime, Shanghai, China). Briefly, cells that had been seeded on six-well plates were homogenized with lysis buffer (1% Triton X-100, 0.1% SDS,150 mM NaCl, 50 mM Tris-HCl pH7.5, and 1% NaDOC) supplemented with a Complete protease cocktail inhibitor (Roche). After the supernatants from the cells were added to the ATP Detection Reaction Solutions from the kit, (which contain 1 mM dithiothreitol, 0.5 mM luciferin, and 12.5 μg/mL luciferase), the intensity readings of the mixtures were obtained via a luminometer on an Infinite^®^ 200 Microplate Reader. The ATP concentrations in the samples were calculated based on an ATP standard curve. The protein concentrations were quantified with a BCA Protein Assay Kit (Beyotime, Shanghai, China) and were used for normalization. The cellular ATP levels were expressed as the percentage of the level in the control group (nmol/mg protein).

### Western blot analysis

Protein samples were extracted from human liver tissues, animal tissues and HCC cell lines, and were separated by SDS-PAGE. After the samples were transferred to PVDF membranes, the membranes were blocked and incubated with various primary antibodies at 4°C overnight. The primary antibodies used in this study are listed in Supplementary Table 1. After the membrane was incubated for one hour with a horseradish peroxidase–conjugated anti-mouse or anti-rabbit secondary antibody, the membrane was visualized with a chemiluminescent detection system (BIO-RAD, Hercules, CA) using the Super Signal West Pico chemiluminescent substrate (Pierce Biotechnology, THP). The relative protein levels were measured with Image Lab^™^ software and a Molecular Imager^®^ ChemiDoc^™^ XRS + System (BIO-RAD).

### Quantitative real-time polymerase chain reaction

Total RNA from liver tissues and transfected cells was extracted with the RNAiso Plus Reagent (Takara Biotechnology, Otsu, Japan), and reverse transcription was performed with the PrimeScript^™^ RT reagent Kit (Takara). The cDNA samples were subjected to quantitative real-time PCR (qRT-PCR) using SYBR^®^ Premix Ex Taq^™^ II (Takara); the reactions were performed on a BIO-RAD CFX96^™^ C1000 Real-Time System. The mtDNA copy numbers and transcript levels were also detected on this device, as described above. The Ct values of the genes were determined, and then relative expression of each gene was quantified and analyzed with BIO-RAD CFX Manager software (BIO-RAD). The specific primers used for PCR in this study are listed in Supplementary Table 2. β-actin and *GAPDH* were chosen as the internal standards for the HCC cell lines and liver tissues, respectively.

### Immunofluorescence staining

To analyze the expression of EMT markers in the HCC cells after SIRT1 downregulation, immunofluorescence staining was performed according to the manufacturer's protocol. Briefly, SIRT1-depleted cells were seeded onto coverslips and permeabilized with 0.25% Triton for 10 minutes. Nonspecific binding sites were blocked with 1% bovine serum albumin (BSA) for 30 minutes. Subsequently, the coverslips were stained with mouse anti-CK18 (Abcam, Cambridge, MA), rabbit anti-vimentin (Santa Cruz Biotechnology Inc.) and mouse anti-fibronectin (Santa Cruz) antibodies overnight, and then incubated with an Alexa Fluor 647-conjugated rabbit anti-mouse IgG or goat anti-rabbit IgG secondary antibody (Invitrogen, Carlsbad, CA). Nuclei were stained with DAPI, and the coverslips were mounted with an anti-fade reagent. Finally, the cells were analyzed under the confocal laser-scanning microscope.

### Immunohistochemistry and *in situ* examination

Immunohistochemical staining was performed on 5-μm sections of paraformaldehyde- fixed, paraffin-embedded tumor samples, adjacent nontumoral liver tissue, normal liver tissue and mouse lung tissue. The details of the immunohistochemistry were described previously [28]. The slides were incubated with mouse anti-SIRT1 (Abcam), rabbit anti-TFAM (Abcam), rabbit anti-COXIV (Cell Signaling Technology, Danvers, MA, USA) and rabbit anti-TOMM20 (Abcam) primary antibodies. The mouse lung tissue sections (5-μm thick) were stained with hematoxylin and eosin (H&E) so that tumor nodules could be observed. The slides were evaluated under a light microscope (Leica, DM6000M).

### Mouse tumorigenicity assay

Subcutaneous injections were carried out so that the proliferative effects of SIRT1 on HCC could be further evaluated. BALB/C nude mice (four weeks old) were housed under standard conditions and cared for according to the institutional guidelines for animal care. Briefly, stably transfected SIRT1-knockdown or vector-control MHCC97H cells (5 × 10^6^) were suspended in PBS and subcutaneously inoculated into the rear flanks of nude mice. The mice were monitored every three days and tumors were measured weekly with calipers. The final tumor weights and volumes were measured four weeks later, after the mice had been sacrificed.

### *In vivo* metastasis assay

Orthotopic implantation and a tail vein injection assay were conducted to assess the effect of SIRT1 on tumor metastasis. For the orthotopic implantation, 4.0 × 10^6^ MHCC97H-sh-*SIRT1* (hU6-MCS-Ubiquitin-*Luc_firefly*-IRES-puromycin), MHCC97H-vector (hU6-MCS-Ubiquitin-*Luc_firefly*-IRES-puromycin) and MHCC 97H-sh-*SIRT1*+ *PGC-1a*-cDNA (hU6-MCS-Ubiquitin-*Luc_firef*ly-IRES-puromycin) cells were suspended in 100 μL of DMEM and Matrigel (Corning, Lifesciences, Bedford, MA) (1:1) and then inoculated into the liver parenchyma of nude mice under 7% chloral hydrate anesthesia. The mice were monitored every two days and sacrificed six weeks later. The liver and lung tissues from the mice were examined histopathologically.

For the tail vein injection, 1 × 10^6^ SIRT1-knockdown or sh-control MHCC97H cells in 200 μL of PBS were injected into the lateral tail veins of four-week-old BALB/C nude mice. After six weeks, the mice were sacrificed and the tumor nodules that had formed on the surfaces of the lungs and livers were counted. The lungs and livers were perfused with 0.9% saline, fixed with 4% paraformaldehyde and excised for further study.

### Bioluminescence imaging and analysis

The nude mouse model of pulmonary metastasis was established via orthotopic implantation and tail vein injection. For *in vivo* tracking, different groups of cells were transfected with a lentivirus containing firefly luciferase. Tumor formation and metastasis were imaged by bioluminescence at the indicated times. Briefly, the mice were anesthetized with 3% isoflurane after intraperitoneal administration of 150 mg/kg body weight of a D-luciferin potassium salt (Bridgen, Beijing, China) for imaging. Bioluminescence was detected with an IVIS Spectrum System 100 (Perkin Elmer, Waltham, MA). Fifteen minutes after injection with the D-luciferin potassium salt, the mice were photographed at various exposure times (1–300 seconds). The achieved grayscale photographic and pseudo-colored luminescent images were automatically superimposed by IVIS Living Image software (Perkin Elmer), so that the observed luciferase signal and its location in the mouse could be analyzed. The data from the fluorescence images were acquired and analyzed with Living Image software (Perkin Elmer).

### Statistical analysis

Quantitative comparisons of the relative SIRT1 levels between HCC tissue and adjacent nontumoral liver tissues were analyzed with a paired Student's *t* test. The correlations between SIRT1 expression and individual clinicopathological parameters were evaluated with a nonparametric chi-squared test, Spearman's rank test, and Student's *t* test. The overall and disease-free survival rates were calculated from the date of tumor resection to the time of first recurrence (disease-free survival) or death (overall survival), and Kaplan-Meier analysis was used to estimate the survival rates according to SIRT1 expression. The data among the groups were compared with one-way ANOVAs. All experimental data were expressed as the mean ± SEM, and all statistical analyses were performed with SPSS software (version 13.0). A two-tailed *P* value < 0.05 was regarded as statistically significant.

## SUPPLEMENTARY MATERIALS TABLES


