# Supplementary material for: SIRT1 facilitates hepatocellular carcinoma metastasis by promoting PGC-1α-mediated mitochondrial biogenesis
Source: Oncotarget. 2016 Apr 12;7(20):29255–74. doi: 10.18632/oncotarget.8711 (PMC5045394; doi:10.18632/oncotarget.8711)
Supplement: Supplementary file 1 [file oncotarget-07-29255-s001.pdf]

SIRT1 facilitates hepatocellular carcinoma metastasis by promoting PGC-1α-mediated mitochondrial biogenesis

Supplementary Materials

SupplementaryTable S1: The information of antibodies used in this study

Antibodies	Company	Clone	Product Number	Dilution immunoblot	positive location
SIRT1	Abcam	19A7AB4	ab110304	1:8000	Nuclear and/or Cytoplasm
E-cadherin	Abcam	HECD-1	ab1416	1:1000	Cell membrane
Cytokeratin 18	Santa Cruz	DC-10	sc-6259	1:500	Cytoplasm
Vimentin	Santa Cruz	H-84	sc-5565	1:400	Cytoplasm
α-SMA	Abcam	E184	ab32575	1:1000	Cytoplasm
Twist	GeneTex	10E4E6	GTX60766	1:1000	Nuclear and/or Cytoplasm
Snail	Santa Cruz	H-130	sc-28199	1:200	Nuclear and/or Cytoplasm
Fibronectin	Santa Cruz	EP-5	sc-8422	1:400	Cytoplasm
Tfam	cell signalling	D5C8	#8076	1:1000	Mitochondria
COX IV	cell signalling	3E11	#4850	1:1000	Mitochondrial inner membrane
TOMM20	Abcam	4F3	ab115746	1:1000	Mitochondrial outer membrane
PGC-1α	Santa Cruz	H-300	sc-13067	1:200	Cytoplasm and/or Nuclear
β-actin	Sigma Aldrich	AC-15	A1978	1:5000	Cytoplasm

Supplementary Table S2: Primer sequences used in this study

SIRT1	Forward	5′-GCTCGCCTTGCTGTAGACTTCC-3′
	Reverse	5′-ACCTGTTCCAGCGTGTCTATGTTC-3′
E-cadherin	Forward	5′-TTCTGCTGCTCTTGCTGTTTCTTC-3′
	Reverse	5′-CCTCTTCTCCGCCTCCTTCTTC- 3′
Vimentin	Forward	5′-CACCAGCCGCAGCCTCTAC-3′
	Reverse	5′-CGAGAAGTCCACCGAGTCCTG-3′
Fibronnectin	Forward	5′-GCGGAGAGCAGCGAAGAAGG-3′
	Reverse	5′-GCGGCGGAGGAGGAGGAG-3′
Twist	Forward	5′-GGCACCATCCTCACACCTCTG-3′
	Reverse	5′- GCTGATTGGCACGACCTCTTG-3′
Snail	Forward	5′-ACCGCCTCGCTGCCAATG-3′
	Reverse	5′-GCCTTTCCCACTGTCCTCATCTG-3′
mtDNA copy number (probe)	Forward	5′-CAAACCTACGCCAAAATCCA-3′
	Reverse	5′-GAAATGAATGAGCCTACAGA-3′
COX I	Forward	5′-ACTAACAGACCGCAACCTCAACA-3′
	Reverse	5′-CCGAAGCCTGGTAGGATAAGAAT-3
ND1	Forward	5′-CTAATCGCAATGGCATTCCTAA-3′
	Reverse	5′-TGGTAGATGTGGCGGGTTTT-3′
ND6	Forward	5′-AAAGTTTACCACAACCACCACCC-3′
	Reverse	5′-ATTGAGGAGTATCCTGAGGCATG-3′
β-actin	Forward	5′-GTGAAGGTGACAGCAGTCGGTT-3′
	Reverse	5′-GAAGTGGGGTGGCTTTTAGGA-3′
GAPDH	Forward	5′-TGACAACAGCCTCAAGAT-3′
	Reverse	5′-GAGTCCTTCCACGATACC-3′
